# On infantile colic: Comments on the article by Skjeie and coworkers

**DOI:** 10.3109/02813432.2014.892303

**Published:** 2014-03

**Authors:** Oscar Heyerdahl, Nils Lystad

**Affiliations:** Huldreveien 2 B 0276 Oslo, Norway. E-mail: nlystad@online.no

**Dear Sir**

We would like to congratulate Skjeie and coworkers on their well-accomplished study [1], but still, we would like to make some comments.

The authors do not comment on the marked reduction in total crying time in both the acupuncture group and the control group already on the third of the three consecutive treatment days. The acupuncture group had a somewhat faster reduction, but the difference was not statistically significant. On the fourth day after baseline, the daily crying in the acupuncture group was reduced from 220 minutes to 120 minutes. We think that a 45% reduction in crying time would be most welcome to tired and worried parents. On the fourth day from the start of treatment, only nine out of 38 in the acupuncture group and 10 out of 41 in the control group still met Wessel's criteria for infantile colic.

An explanation for this reduction could be that it represents the natural course of the ailment. This we do not know, as there was no completely untreated group in the study. The very steep curve displaying the reduction in crying time in immediate connection with the treatment does not resemble what would be expected from a natural fading out of the disease.

Another hypothetical cause could be a placebo effect on the parents.

A third possibility could be that both groups have had sensory stimulation on point ST 36.

1. The acupuncture group was given a mark on ST 36 with a felt pen, an acupuncture needle, and a small adhesive bandage that was removed the next day to cover any possible needle mark. There was no manipulation of the needle, which was retained for only 30 seconds. There is no clear knowledge as to what is sufficient acupuncture stimulation in an infant. The needling procedure in the study would be considered as a minimal stimulus.
2. The control group was given the same procedure, except for the needling. One of us, NL, participated in the study and observed that many of the children cried when the adhesive bandage was removed. The children's reaction to the bandage removal seemed stronger than their reaction to the needling, which might indicate that the removal of the adhesive bandage evoked at least a similar level of sensory stimulus as the needling itself.

It might well be that the lack of significant difference between the two groups is due to the adhesive bandage procedure. This study might thus be a comparison between two groups that both received sensory stimulation. A possible suboptimal needling procedure might have added to the lack of significant difference between the two groups.

It would be interesting if the authors would comment on the reduction in crying time discussed above and also reflect on the difference between their results and the results of the two Swedish RCT studies that found significant differences between the acupuncture group and control group on several parameters.
